# RIPK3-Mediated Necroptosis and Neutrophil Infiltration Are Associated with Poor Prognosis in Patients with Alcoholic Cirrhosis

**DOI:** 10.1155/2018/1509851

**Published:** 2018-11-25

**Authors:** Zhenzhen Zhang, Guomin Xie, Li Liang, Hui Liu, Jing Pan, Hao Cheng, Hua Wang, Aijuan Qu, Yan Wang

**Affiliations:** ^1^Department of Infectious Diseases, Peking University First Hospital, Beijing 100034, China; ^2^Xiamen Branch, Zhongshan Hospital, Fudan University, Xiamen 361015, China; ^3^Department of Physiology and Pathophysiology, School of Basic Medical Sciences, Capital Medical University, Key Laboratory of Remodeling-Related Cardiovascular Diseases, Ministry of Education, Beijing Key Laboratory of Metabolic Disturbance-Related Cardiovascular Disease, Beijing 100069, China; ^4^Department of Pathology, Peking University First Hospital, Beijing 100034, China; ^5^Department of Pathology, Beijing Youan Hospital, Capital Medical University, Beijing 100069, China; ^6^Department of Oncology, The First Affiliated Hospital, Institute for Liver Diseases of Anhui Medical University, Hefei 230032, China

## Abstract

Alcoholic cirrhosis is an end-stage liver disease with impaired survival and often requires liver transplantation. Recent data suggests that receptor-interacting protein kinase-3- (RIPK3-) mediated necroptosis plays an important role in alcoholic cirrhosis. Additionally, neutrophil infiltration is the most characteristic pathologic hallmark of alcoholic hepatitis. Whether RIPK3 level is correlated with neutrophil infiltration or poor prognosis in alcoholic cirrhotic patients is still unknown. We aimed to determine the correlation of RIPK3 and neutrophil infiltration with the prognosis in the end-stage alcoholic cirrhotic patients. A total of 20 alcoholic cirrhotic patients subjected to liver transplantation and 5 normal liver samples from control patients were retrospectively enrolled in this study. Neutrophil infiltration and necroptosis were assessed by immunohistochemical staining for myeloperoxidase (MPO) and RIPK3, respectively. The noninvasive score system (model for end-stage liver disease (MELD)) and histological score systems (Ishak, Knodell, and ALD grading and ALD stage) were used to evaluate the prognosis. Neutrophil infiltration was aggravated in patients with a high MELD score (≥32) in the liver. The MPO and RIPK3 levels in the liver were positively related to the Ishak score. The RIPK3 was also significantly and positively related to the Knodell score. In conclusion, RIPK3-mediated necroptosis and neutrophil-mediated alcoholic liver inflammatory response are highly correlated with poor prognosis in patients with end-stage alcoholic cirrhosis. RIPK3 and MPO might serve as potential predictors for poor prognosis in alcoholic cirrhotic patients.

## 1. Introduction

Alcoholic cirrhosis is the end-stage serious liver disease with high morbidity and mortality and is the leading cause of liver transplantation [1–3]. Prognostic models can be used to assess the severity and survival of the disease and can be useful as a medical decision-making tool to guide patient care. However, the early detection and evaluation of this severe disease have not been fully elucidated.

The most widely used noninvasive predictor of poor prognosis in alcoholic liver cirrhosis is the model for end-stage liver disease (MELD) scoring systems [4]. While for specific predictors, histologic scoring system is the main method [5, 6]. The ALD grading and staging schema were first proposed for alcoholic liver disease [7], then recently the alcoholic hepatitis histologic score (AHHS), proposed for alcoholic hepatitis (AH) [8]. And the Ishak score [9] and Knodell score [10] were recognized predictors for chronic hepatitis. However, these scoring systems are based on clinical, biochemical, and histological features and do not take into consideration the molecular pathogenesis of the disease. Thus, identification of pathogenesis-related factors indicating poor prognosis in patients with alcoholic cirrhosis is necessary for early prevention and treatment.

The pathogenesis of alcoholic cirrhosis is characterized by inflammation, fibrosis, and damaged cellular membranes incapable of detoxification ending in scaring and necrosis [11–13]. Recently, it has been reported that necroptosis, i.e., programmed cell necrosis, plays an important role in alcoholic cirrhosis [14, 15]. Receptor-interacting protein kinase 3 (RIPK3) is a key component of the necrosome [16, 17] and was reported to be activated in the livers of mouse models after chronic ethanol feeding as well as in the livers of ALD patients [18]. Furthermore, RIP3-knockout mice could prevent ethanol-induced liver injury and inflammation [18]. RIPK3 has been shown to interact with RIPK1 in kinase activation [19]. However, in some circumstances, RIPK3 serves its necrotic role independent of RIPK1 in viral infection [20], in TNF-*α* mediated shock [21], and also in ethanol-induced liver injury [18]. Whether the expression of RIPK3 is related to a poor prognosis in alcoholic cirrhosis is hitherto unknown.

Neutrophil infiltration is another pathologic hallmark for alcoholic cirrhotic liver [22]. Our previous studies demonstrate that neutrophil infiltration plays a major role in alcoholic liver disease of murine models [23, 24]. Neutrophil depletion by a pharmacological agent (anti-Ly6G antibody) ameliorates alcoholic liver injury [23]. It is also reported that the expression of CXC chemokines recruiting the neutrophil infiltration in the liver is associated with the prognosis of patients with alcoholic hepatitis [25], although several studies have indicated that programmed cell death may trigger inflammation in liver diseases of murine models [16], such as viral infection [20], systemic inflammatory response syndrome or sepsis [26], drug-induced liver injury [27], and alcoholic liver disease [18, 28]. However, whether RIPK3 level is correlated with neutrophil infiltration in alcoholic cirrhotic patients is still unknown.

In the present study, we aimed to determine the correlation between RIPK3 with the degree of neutrophil infiltration in the liver and the related prognosis in the end-stage alcoholic cirrhotic patients. Our results showed that RIPK3-mediated necroptosis and neutrophil-mediated alcoholic liver inflammatory response are highly correlated with poor prognosis in end-stage alcoholic cirrhotic patients. RIPK3 and MPO may serve as potential predictors for poor prognosis in patients with alcoholic cirrhosis.

## 2. Materials and Methods

### 2.1. Patients

In this retrospective study, a total of 20 diagnosed alcoholic cirrhotic patients (stage 3 to 4 fibrosis according to clinical practice guidelines [3, 29]) and 5 healthy controls were analyzed during liver transplantation from the Liver Tissue Cell Distribution System, University of Minnesota (Minneapolis, MN), between 2006 and 2011 [30]. Patients with concomitant other causes of liver disease, including chronic hepatitis B, chronic hepatitis C, hepatocellular carcinoma and nonalcoholic fatty liver disease, autoimmune liver disease, and drug-induced liver injury, were excluded.

### 2.2. Data Collection

Clinical and biochemical parameters at the time of liver transplantation were carefully collected from the medical records. The MELD scores were calculated. Then the patients were divided into two groups based on a MELD score greater than or less than 32 [31]. The liver tissue was fixed in 10% formalin and paraffin-embedded for histological evaluation. All patients provided written consent, and the protocol was approved by the clinical research ethics committee of the Liver Tissue Cell Distribution System, University of Minnesota (Minneapolis, MN), and executed according to the Declaration of Helsinki.

### 2.3. Histological Assessment

Deparaffinized liver sections (5 *μ*m thick) were stained with hematoxylin and eosin (H&E) and immunohistochemical staining for MPO, RIPK1, RIPK3, and pMLKL using the DAB kit (Gene Tech, China) according to the manufacturer's protocol. The primary antibodies used were anti-myeloperoxidase (MPO) (Biocare Medical, Concord, CA), anti-RIPK3 (WuXi App Tec, China), anti-RIPK1 (Cell Signaling, USA), and pMLKL (Abcam, USA). Terminal deoxynucleotidyl transferase-mediated deoxyuridine triphosphate nick-end labeling (TUNEL) staining was performed with an ApopTag Peroxidase In Situ Cell Death Detection Kit (Roche, Mannheim, GER). All slides were evaluated by two pathologists blinded to patient clinical information. MPO- and RIPK3-positive cells were quantified randomly from 5 fields at 100x magnification per patient using Image J software 1.46r (NIH, USA). The corresponding histological features of ALD grading and staging schema [7], Ishak score [9], Knodell score/histology activity index [10], and alcoholic hepatitis histologic score (AHHS) [8] were numerically evaluated.

### 2.4. Statistical Analysis

The statistical analyses were performed between the two groups. All data were treated as continuous variables. For the data conformed to the normal distribution, the average and standard error of the mean (SEM) were displayed using Student's *t*-test. For nonnormality, the median and interquartile ranges were analyzed and compared using Wilcoxon rank-sum (Mann–Whitney) tests. The correlation analysis was using Pearson's correlation test. All calculations were performed using SPSS 23.0 (IBM, Armonk, NY), and *P* < 0.05 was considered significant.

## 3. Results

### 3.1. Neutrophil Infiltration Is a Hallmark of Alcoholic Cirrhosis

**Figure 1:**
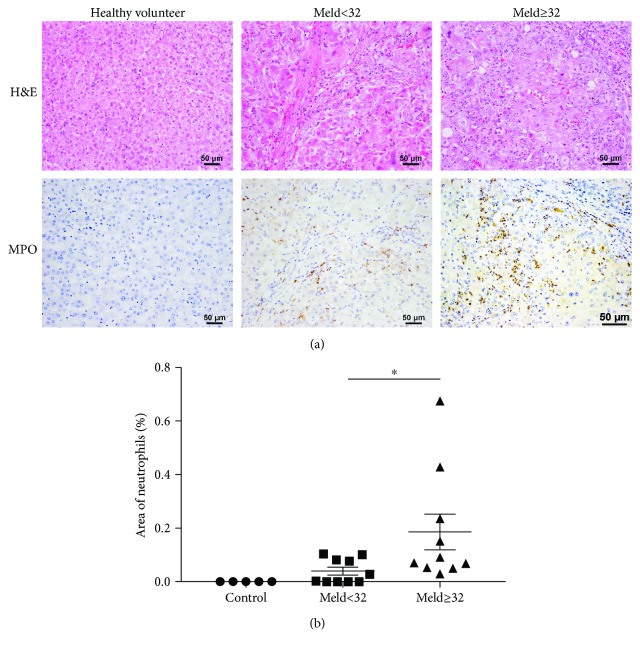
Hepatic neutrophil infiltration is a hallmark of alcoholic cirrhosis. Representative images of H&E staining (a) and immunohistochemistry (IHC) of MPO (b) were examined in patients with end-stage alcoholic cirrhosis and healthy controls. Data were represented as the means ± SEM. ^∗^*P* < 0.05.

Inflammatory infiltration is one of the hallmarks for alcoholic cirrhosis [23, 24]. To confirm whether neutrophil infiltration contributes to this process, H&E staining and IHC staining for MPO were performed. As shown by H&E staining in Figure 1(a), there were obvious inflammatory foci around the necrotic area in the liver of cirrhosis patients but not in healthy volunteers. IHC staining for the neutrophil marker MPO demonstrated that the inflammatory foci were mainly MPO-positive neutrophils (Figure 1(b)). This is consistent with previous studies [11, 23–25, 32] indicating that neutrophil infiltration plays an important role in alcohol-induced liver injury.

### 3.2. RIPK3, But Not RIPK1, Is Highly Expressed in Patients with Alcoholic Cirrhosis

**Figure 2:**
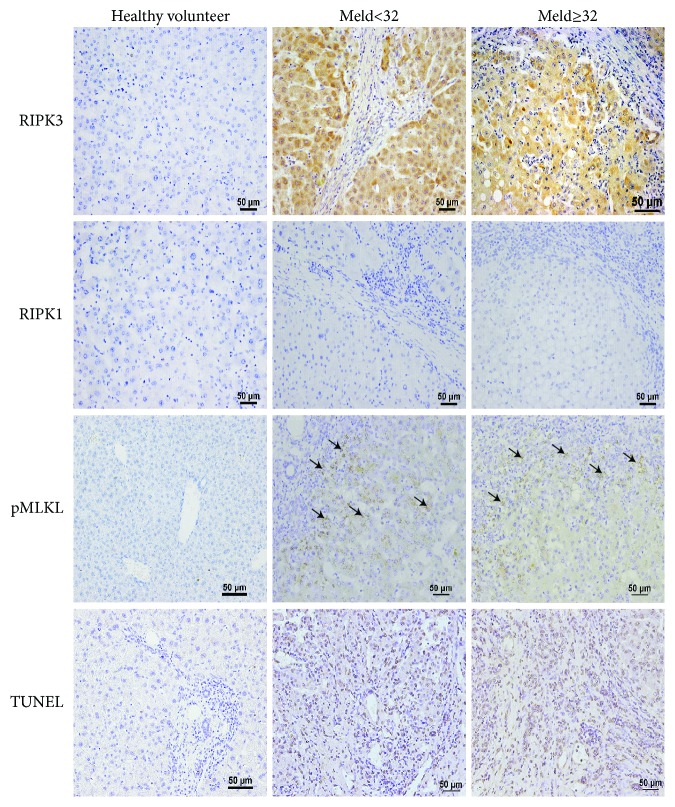
RIPK3, but not RIPK1, is highly expressed in patients with alcoholic cirrhosis. Representative images of immunohistochemistry (IHC) of RIPK1, RIPK3, pMLKL, and TUNEL staining were examined in patients with end-stage alcoholic cirrhotic patients and healthy controls. *n* = 20.

Alcoholic cirrhosis is associated with necrotic hepatocyte cell death, called necroptosis [14], which is regulated by RIP1-RIP3-MLKL- (mixed lineage kinase domain-like protein-) mediated necrotic cascade, but the role of RIPK1 and RIPK3 in the pathogeneses of alcoholic liver cirrhosis is largely unknown. To further investigate whether RIPK1 or RIPK3 mediates the pathogeneses of alcoholic liver cirrhosis, IHC analyses of RIPK1 and RIPK3 in patients with alcoholic cirrhosis were examined. As shown in Figure 2, very strong RIPK3 staining but not RIPK1 was found in the necrotic area, indicating that RIPK3, but not RIPK1, was involved in alcoholic liver cirrhosis, consistent with previous studies showing RIPK3 is mediated in the mouse model of ethanol-induced liver injury and progression of ALD patients [27]. Furthermore, phospho-mixed lineage kinase-like protein (p-MLKL) a downstream molecule of RIPK3 in necroptosis pathway was also activated in the necrotic area in patients with alcoholic cirrhosis. Finally, the increased cell death in alcoholic cirrhotic patients was further confirmed by TUNEL staining.

### 3.3. Neutrophil Infiltration and the level of RIPK3 Are Associated with Poor Prognosis Based on MELD over 32

To explore whether the neutrophil infiltration or the level of RIPK3 can predict prognosis according to MELD over 32 or not, patient clinical parameters are measured to assess liver function on the day of enrollment to liver transplantation and the prognosis scores of histologic parameters according to MELD greater or less than 32 are summarized in Table 1. The results revealed that patients with MELD score over than 32 were associated with upregulation of the levels of MPO, indicating they can be used as indicators of poor prognosis.

### 3.4. Neutrophil Infiltration and the Expression of RIPK3 Are Associated with Poor Prognosis Based on Histologic Parameters

**Figure 3:**
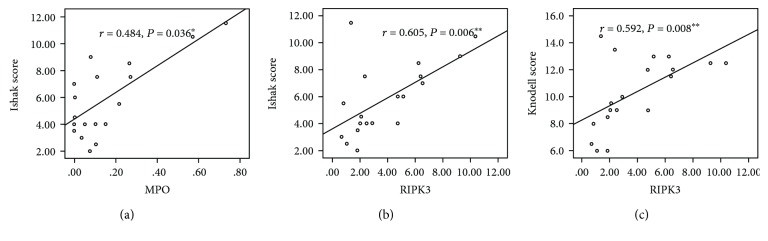
Correlation between RIPK3 and MPO with histological scoring systems. The correlation analyses of RIPK3 and MPO with histological scoring systems were examined at those alcoholic cirrhotic patients. Data were represented as the means ± SEM. ^∗^*P* < 0.05 and ^∗∗^*P* < 0.01.

To further investigate the relationship between RIPK3 expression, neutrophil infiltration, and histologic parameters, correlation analysis was performed. The prognostic histological score systems, including Ishak score [9], Knodell score [10], ALD grading and staging schema [7], and alcoholic hepatitis histologic score (AHHS) [8], were analyzed by two pathologists. As shown in Figure 3, significant correlations were observed between the Ishak score and the area of MPO and RIPK3 staining in patients' livers (*r* = 0.484 and *P* = 0.036 and *r* = 0.605 and *P* = 0.006, respectively). Importantly, the RIPK3 was also significantly and positively related to the Knodell score (*r* = 0.592, *P* = 0.008).

## 4. Discussion

The current study investigated the relationship between RIPK3-mediated necroptosis and neutrophil-mediated alcoholic liver inflammation with disease prognosis. The results demonstrated that MELD (a widely recognized noninvasive predictor of disease outcome) and histological prognostic scores (the invasive predictor) were well correlated to the levels of neutrophil infiltration and the expression of RIPK3. Importantly, RIPK3 and MPO may act as factors to predict poor outcomes in patients with alcoholic cirrhosis.

One of the most intriguing features in alcoholic cirrhosis is the remarkable hepatic neutrophil infiltration. Neutrophil infiltration has played an important role in promoting the development of alcoholic cirrhosis in murine models [23, 24, 32]. Either pharmacological inhibition or genetic deletion of E-selectin [23], an important adhesion molecule for neutrophil migration, or CXCL1 [24], a key chemokine in neutrophil recruitment, can prevent mice from ethanol-induced hepatic neutrophil infiltration. Therefore, there is an urgent need for further translational studies using human samples to identify neutrophil targets for therapy. Here, the pathogenic role of neutrophil infiltration was shown in alcoholic cirrhotic patients.

Another essential finding from this study is the meaningful confirmation of RIPK3 but not RIPK1 as being significantly upregulated in human livers. RIPK1 and RIPK3 are recently discovered proteins that regulate caspase-independent programmed cell death, called necroptosis [16, 17, 33]. RIPK3 is strongly expressed in the alcoholic cirrhotic patients in our study, and the pMLKL, which is a substrate for RIPK3 kinase activity [16, 17], was also activated in the necrotic area. Roychowdhury et al. first reported elevated RIPK3 expression in ethanol-induced liver injury murine models and in human ALD samples independent of RIPK1. A deficiency of RIPK3 extremely reduced the severity of ethanol-induced liver injury, but not for RIPK1-specific inhibitor [18]. This mechanism is used because of impaired hepatic proteasome function failing to produce RIPK3, as pharmacological inhibition or genetic disruption of proteasome accumulates RIPK3. However, another study also showed that RIPK1 decreased Gao-binge-induced neutrophil infiltration [34]. It remains a controversial issue whether RIPK1 or RIPK3 is correlated with neutrophil infiltration and needs to be further studied. Our study showed that RIPK3 but not RIPK1 was activated in patients with alcoholic cirrhosis. Further studies using liver-specific RIPK3 KO and RIPK1 KO mice should be conducted to confirm these results.

To show the prognostic value of neutrophil infiltration and RIPK3 in patients with alcoholic cirrhosis, the noninvasive prognostic score MELD and the invasive histological scoring system were evaluated. The MELD score is a recognized prognosis predictor in liver cirrhosis, especially for those waiting for liver transplantation [31, 35]. The results of our study show that neutrophil infiltration was upregulated in the group with a MELD score greater than 32, indicating neutrophil infiltration may represent poor prognosis in alcoholic cirrhosis. This is consistent with previous findings that neutrophil infiltration may promote the development of alcoholic cirrhosis [23, 24, 32].

On the other hand, the histological scoring systems predicting ALD have not been uniform. Yip and Burt first recommended a grading and staging scoring system for the assessment of histological severity of ALD in 2006 [7]. It was verified recently that even the early or compensated ALD should be evaluated as a predictor of long-term mortality [36]. Altamirano et al. proposed an AHHS scoring system using AH to predicting patients' outcomes [8]. However, this study excluded the other spectrum of ALD except for AH, and whether AHHS applies to those patients remains unknown. The patients in our study all had end-stage alcoholic cirrhosis, and the results of our study between the relationship of the AHHS score and clinical parameters, MPO, or RIPK3 were not significant, suggesting the AHHS score may not be suitable for alcoholic cirrhosis. This should be further validated by a more prospective study.

The Knodell score and Ishak score are frequently used in chronic hepatitis, particularly HCV [9, 10]. The position of fibrosis differs between HCV and ALD, because HCV begins with a periportal distribution of fibrosis and extending to the portal center, whereas ALD starts with central expansion [29]. This means that there will be more fibrosis in ALD patients than HCV patients in the early stage of the disease. However, because patients in our study all have end-stage alcoholic cirrhosis (fibrosis score more than 4), which eliminated this difference, so the Ishak score and Knodell score were used to assess the patient histological features. The results show that MPO and RIPK3 correlated to the Ishak score and RIPK3 correlated to the Knodell score, suggesting MPO and RIPK3 may be good prognostic factors for the patients' outcome based on histological parameters.

Furthermore, the following limitations of this study need to be considered. Firstly, this is a retrospective study observing patient prognostic indicators and the sample size is limited due to the difficulty in obtaining samples. Further prospective studies containing larger samples should be performed to confirm this finding. Second, all the patients underwent liver transplantation; therefore, disease mortality could not be directly evaluated, so prognostic indicators were analyzed relative to prognostic MELD models and histologic parameters. Clearly, these findings should be confirmed in further prospective studies analyzing the mortality during hospitalization and the medium and long-term (admission, 30 days to 3 months, or 6 months, respectively) outcome and compare the different scores with analytic parameters or others.

## 5. Conclusions

The present study demonstrates that RIPK3 and neutrophil infiltration in patients with alcoholic cirrhosis can be used to predict poor disease prognosis based noninvasive predictors MELD and invasive histological scoring systems.

## Figures and Tables

**Table 1:** Comparative analysis of patients with MELD greater or less than 32.

	MELD ≥ 32 (*n* = 10)	MELD < 32 (*n* = 10)	*t*/*Z* value	*P* value
MPO (%)	0.0806 (0.05195, 0.2874)	0.0151 (0.00, 0.08675)	−2.125	0.034^∗^
RIPK3 (%)	4.7367 (1.7054, 6.2929)	2.0914 (1.4491, 4.7188)	−0.735	0.462
Ishak score	6.75 (5.125, 9.000)	4.00 (3.25, 5.75)	−1.847	0.065
Ishak fibrosis score	6.00 (6.00, 6.00)	6.00 (5.5, 6.0)	−0.860	0.390
Knodell score	11.3 ± 2.7305	9.222 ± 2.1667	−1.823	0.086
AHHS score	6.70 ± 1.11056	6.3889 ± 1.21906	−0.582	0.568
ALD grading	6.90 ± 2.3781	5.556 ± 1.5899	−1.431	0.171
ALD stage	6.00 (5.00, 6.00)	6.00 (5.00, 6.00)	−0.159	0.874

MPO: myeloperoxidase; RIPK3: receptor-interacting protein kinase3; MELD: model for end-stage liver disease; AHHS: alcoholic hepatitis histologic score. ^∗^*p* < 0.05.

## Data Availability

The data used to support the findings of this study are available from the corresponding author upon request.
